# Image-based phenotyping to estimate anthocyanin concentrations in lettuce

**DOI:** 10.3389/fpls.2023.1155722

**Published:** 2023-04-03

**Authors:** Changhyeon Kim, Marc W. van Iersel

**Affiliations:** ^1^ Department of Horticulture and Crop Science, The Ohio State University, Columbus, OH, United States; ^2^ Department of Horticulture, The University of Georgia, Athens, GA, United States

**Keywords:** anthocyanins, remote sensing, anthocyanin index, non-destructive measurement, low-cost plant phenotyping, controlled environment agriculture (CEA)

## Abstract

Anthocyanins provide blue, red, and purple color to fruits, vegetables, and flowers. Due to their benefits for human health and aesthetic appeal, anthocyanin content in crops affects consumer preference. Rapid, low-cost, and non-destructive phenotyping of anthocyanins is not well developed. Here, we introduce the normalized difference anthocyanin index (NDAI), which is based on the optical properties of anthocyanins: high absorptance in the green and low absorptance in the red part of the spectrum. NDAI is determined as (I_red_ - I_green_)/(I_red_ + I_green_), where I is the pixel intensity, a measure of reflectance. To test NDAI, leaf discs of two red lettuce (*Lactuca sativa*) cultivars ‘Rouxai’ and ‘Teodore’ with wide range of anthocyanin concentrations were imaged using a multispectral imaging system and the red and green images were used to calculate NDAI. NDAI and other commonly used indices for anthocyanin quantification were evaluated by comparing to with the measured anthocyanin concentration (n = 50). Statistical results showed that NDAI has advantages over other indices in terms of prediction of anthocyanin concentrations. Canopy NDAI, obtained using multispectral canopy imaging, was correlated (n = 108, R^2^ = 0.73) with the anthocyanin concentrations of the top canopy layer, which is visible in the images. Comparison of canopy NDAI from multispectral images and RGB images acquired using a Linux-based microcomputer with color camera, showed similar results in the prediction of anthocyanin concentration. Thus, a low-cost microcomputer with a camera can be used to build an automated phenotyping system for anthocyanin content.

## 1 Introduction

Anthocyanins are water-soluble pigments that provide red, purple, or blue color to leaves, fruits, and flowers. Anthocyanins accumulate in response to various abiotic and biotic stresses and provide protection to plants against these stressors (Boldt et al., 2014; Liu et al., 2018). Benefits of anthocyanins for human health include anti-cancer activity and alleviating cardiovascular disease and diabetes (Khoo et al., 2017). Due to their benefits and aesthetic appeal, anthocyanins can influence consumer preference. Therefore, rapid phenotyping for anthocyanin content is important in horticultural production and breeding programs, as well as in ecophysiological studies.

Non-destructive estimation of traits using imaging is an ideal approach to phenotyping. Image-based plant phenotyping quantifies reflected light from plants in a non-destructive manner, while the reflected light has unique spectral responses to plant pigments. Multispectral or hyperspectral imaging sensors detect reflected light from plants, and that information is stored as pixels with spatial and quantitative information regarding reflected light intensity and color. Image-based phenotyping can provide reliable data to characterize traits of interest in a non-destructive, rapid, and high-throughput manner, without sampling bias (Del Valle et al., 2018; Costa et al., 2019). These advantages led to an emergence of image-based phenotyping in the early 2010s and many researchers have adopted such systems to screen for traits of interest (Das Choudhury et al., 2019; Maes and Steppe, 2019).

Reflectance imaging can be applied at various scales, including satellites, and plant phenotyping has benefitted from prior work using satellite imaging. Commonly, reflectance indices are calculated using normalization equations, which constrain the index values between -1 and 1. Common indices to quantify vegetation cover, plant health, or physiological status include the normalized difference vegetation index (NDVI) and photochemical reflectance index (PRI) (Rouse et al., 1974; Gamon et al., 1997). Such indices are derived from changes in reflectance based on canopy size or the nutritional and/or physiological status of plants.

Prior work resulted in indices to estimate anthocyanin concentrations: the red to green ratio (Gamon and Surfus, 1999), anthocyanin reflectance index (Gitelson et al., 2001), and modified anthocyanin reflectance index (Gitelson et al., 2006). Bayle et al. (2019) reported the normalized anthocyanin reflectance index (NARI), which is a modified version of the anthocyanin reflectance index. These indices are based on the higher absorptance (or lower reflectance) in the green part of the spectrum (500 – 550 nm) of plants with higher anthocyanin concentrations (Neill and Gould, 2000; Gitelson et al., 2001; Merzlyak et al., 2008). These indices also include red or red-edge reflectance to adjust for the presence of chlorophylls. Most of these indices use reflectance in the red edge (700 - 705 nm) for chlorophyll corrections, due to the strong correlation between the reflectance in the red edge and chlorophyll concentration (Gitelson et al., 1996). At the same time, Gitelson et al. (2001) and Gamon and Surfus (1999) also reported a correlation between chlorophyll concentrations and reflectance in a wide range of the red spectrum (600 - 700 nm). Therefore, anthocyanin predictions may be achieved using reflectance in a wide range of the green and red spectrum, which can be easily acquired by low-cost color (RGB) imaging. However, little is known about the feasibility of predicting anthocyanin concentration using reflectance indices with spectral images acquired by low-cost imaging systems.

With the advance in computing power and better imaging sensors, image-based phenotyping tends to use hyperspectral imaging and machine learning algorithms to process high-throughput data. At the same time, the development of simple indices has become less popular. It is understandable that machine learning approaches have become popular in plant science, because the amount of data generated by hyperspectral imaging system is large, so identification of traits of interest based on relatively simple analyses may not be feasible. However, machine learning may not always provide ideal solutions and may result in errors when the conditions during image acquisition differ from those under which machine learning algorithms were developed. Additionally, machine learning-based models cannot provide underlying physiological meaning to these models.

Using hyperspectral imaging systems to quantify phenotypic traits of interest can be useful, but is not always necessary. Hyperspectral imaging can be expensive and simple alternative approaches may make imaging accessible to many more scientists, as well as horticultural producers. For example, image-based phenotyping of anthocyanin concentrations only requires information in the red and green wavebands based on the optical characteristics of anthocyanins and chlorophyll, so hyperspectral imaging may not be necessary. Furthermore, color imaging provides quantitative information on reflectance in the red, green, and blue wavebands, which may satisfy the requirements for anthocyanin prediction. Therefore, we hypothesized that simple color cameras or multispectral imaging systems can provide quantitative information regarding anthocyanin concentrations.

Here, we introduce a new reflectance index, the normalized difference anthocyanin index (NDAI), for predicting anthocyanin concentration using the images of anthocyanin-rich lettuce cultivars. We evaluated the performance of NDAI by comparing it with other anthocyanin indices in use. We developed a Python script to calculate these indices using multispectral images of leaf discs from two red lettuce cultivars, ‘Rouxai’ and ‘Teodore’ and compared those values to measured anthocyanin concentrations. We also tested whether a low-cost RGB camera, connected to a Raspberry Pi microcomputer can be used to determine the NDAI. Our objectives were (1) to evaluate the performance of different indices, including NDAI, in terms of the prediction of the leaf disc anthocyanin concentration, (2) to validate the best performing index for prediction of anthocyanin concentration at the whole plant scale, and (3) to develop a low-cost RGB imaging system that can predict anthocyanin concentrations.

## 2 Materials and methods

### 2.1 Plant materials

Red-leaf lettuce (*Lactuca sativa*) cultivars ‘Rouxai’ and ‘Teodore’ were grown in a walk-in growth chamber from April 14 to May 14, 2021. Seeds were planted in 10 cm square pots containing a soilless substrate (Fafard 2P Mix; Sun Gro Horticulture, Agawam, MA, USA). The plants were subirrigated biweekly with a water-soluble fertilizer (15N-2.2P-12.5K; Peters Excel 15-15-15 Cal-Mag special Fertilizer, ICL Fertilizers, Dublin, OH, USA). The approximate environmental conditions of the walk-in chamber were a photoperiod of 16 hours, photosynthetic photon flux density (*PPFD*) of 250 µmol m^-2^ s^-1^, daily light integral of 14.4 mol m^-2^ d^-1^, a temperature of 20 °C, a vapor pressure deficit of 1.2 kPa, and CO_2_ concentration of 800 µmol mol^-1^. After 30 days, the plants were transferred to growth chambers with temperatures of 4, 12, and 20 °C for 0, 12, 24, or 36 hours to induce a wide range of anthocyanin concentrations. The other environmental conditions in these growth chambers were similar to those of the walk-in cooler. Each combination of the temperature, exposure time, and cultivar was repeated three times with three biological replications.

### 2.2 Sample collection and multispectral imaging

**Figure 1:**
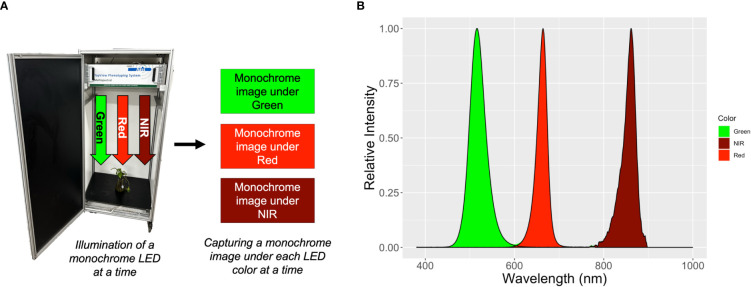
Schematic representation of the acquisition process of green, red, and near-infrared (NIR) spectral images by the TopView multispectral imaging system **(A)** and the normalized spectrum of the LEDs of the system used for capturing each monochrome image **(B)**. The peak and full width at half maximum of green, red, and NIR LEDs were 516 and 40 nm, 664 and 25 nm, and 861 and 28 nm, respectively.

A randomly-selected plant from each exposure time and temperature combination was used for leaf disc imaging and anthocyanin extraction. Two positions on leaves near the top of the plant were selected based on visual assessment of homogeneous anthocyanin distribution. A cardboard piece with a hole with 1-cm radius was clamped on the leaves. The 1-cm exposed leaf discs were imaged using a commercial imaging system (TopView, ARIS, Eindhoven, The Netherlands). To acquire spectral images, the monochrome camera of the imaging system captures one monochrome image at a time under illumination of a monochrome light-emitting diodes (LED) and repeats the process with sequential illumination of seven wavelengths of LED (450, 516, 593, 625, 664, 730, and 861 nm peaks) (**Figure 1A**). We only used the images taken under the green (peak at 516 nm), red (peak at 664 nm), and near infrared (NIR, peak at 861 nm) LEDs (**Figure 1B**), following previous studies of anthocyanin prediction (Gamon and Surfus, 1999; Gitelson et al., 2006; Gitelson et al., 2009; Steele et al., 2009). The full width at half maximum (FWHM) of the green, red, and NIR LEDs was 40, 25 and 28 nm, respectively. During the NIR imaging, the imaging system could not achieve perfect focus. Therefore, image indices using NIR image resulted in a relatively poor spatial resolution (Spectrum of NIR in **Figure 1B**).

Before taking plant images, a middle-gray card (50% reflection at all wavebands) was imaged as a reference to calibrate exposure times of each LED. We measured the pixel intensity of the gray card using Image J software (NIH, Bethesda, MD, USA). Since the middle-gray card should result in an intensity of 127 in a scale of 255 (8-bit resolution of the images), we adjusted the exposure time based on the ratio between 127 and the measured pixel intensity. The calibration with the gray card ensures that images taken under different colors of LEDs are directly comparable and accurate measures of reflectance.

After the multispectral imaging, the 1-cm radius leaf discs were dissected and frozen in liquid nitrogen before anthocyanin extraction and quantification, following a modified protocol of Lee et al. (2005). A leaf disc was weighed and ground with liquid nitrogen. The ground sample was then precipitated in 5 ml of methanol with 2% HCl. After centrifugation, 0.5 ml of the supernatant was transferred to a buffer solution containing 2 ml of 0.025 M KCl (pH 1.0) and 2 ml of 0.4 M sodium acetate (pH 4.5). Absorbance of the solution at 520 and 720 nm was measured using a spectrophotometer (GENESYS 10S UV-Vis spectrophotometer, Thermo Scientific™, Waltham, MA, USA) with three subsamples per the solution. The measured absorbance and the fresh weight (FW) were then used to compute the anthocyanin concentration of the leaf disc per unit FW. A total of 50 leaf discs were used for anthocyanin extraction to evaluate the performance of NDAI and other indices from literatures regarding prediction of anthocyanin concentration.

To test feasibility of predicting anthocyanin concentration in canopy layer using a best performing index in the leaf discs, the intact canopy of 108 lettuce plants was also imaged using the TopView system and used for anthocyanin extraction. Two randomly selected plants from each exposure time and temperature combination were imaged. Since these images only capture the top part of the plant canopy, we collected only the leaves from the top layer of the canopy for anthocyanin extraction following the same protocol. The leaves were ground and homogenized, and approximately 500 mg of the homogenized leaf tissue was used for the extraction.

### 2.3 Anthocyanin index comparisons

The new Normalized Difference Anthocyanin Index (NDAI) is defined as:


(1) $$\text{NDAI}=({\text{I}}_{\text{red}}-{\text{I}}_{\text{green}})/({\text{I}}_{\text{red}}+{\text{I}}_{\text{green}})$$

where I is the pixel intensity, a measure of reflectance, and the subscript indicates the color of the image, while peak and FWHM of the light color vary depending on the light spectrum during imaging. This index uses the same basic equation as the normalized difference vegetation index (Rouse et al., 1974) and photochemical reflectance index (Gamon et al., 1997), which are commonly used normalized indices in remote sensing. We used the green and red wavebands based on the optical properties of anthocyanins *in situ*; higher anthocyanin concentrations result in higher absorptance in the green part of the spectrum, while the absorptance in the red part of the spectrum is related to chlorophyll content (Lichtenthaler, 1987; Gitelson et al., 2001). Therefore, we speculate that NDAI has an advantage over indices that do not use the red part of the spectrum by accounting for chlorophyll content. Indices that use NIR, instead of red, cannot do so.

Bayle et al. (2019) developed the normalized anthocyanin reflectance index (NARI):


(2) $$\text{NARI}=({\left({\text{R}}_{550}\right)}^{-1}-{\left({\text{R}}_{700}\right)}^{-1})/({\left({\text{R}}_{550}\right)}^{-1}+{\left({\text{R}}_{700}\right)}^{-1}$$

where R is reflectance, and the subscript indicates the wavelength (nm). The equation of NARI is mathematically identical to the NDAI. However, the NARI requires narrow green (550 nm) and red edge (700 nm) reflectance that can be acquired from satellite imagery. In contrast, the NDAI uses a wide range of green and red wavebands in the index calculation. We did not include NARI in the evaluation of the performance of various indices, since our equipment does not measure red-edge reflectance and substituting red for red-edge makes NARI identical to NDAI.

To assess the performance of NDAI in the prediction of anthocyanin content, we compared it to previously-developed indices. Gitelson et al. (2001) suggested the anthocyanin reflectance index (ARI):


(3) $$\text{ARI}={\left({\text{R}}_{550}\right)}^{-1}-{\left({\text{R}}_{700}\right)}^{-1}$$

where R is reflectance, and the subscript indicates the wavelength (nm). The ARI described anthocyanin concentrations in maple (*Acer platanoides*), dogwood (*Cornus alba*), geranium (*Pelargonium zonale*), and cotoneaster (*Cotoneaster alaunica*) leaves, with a coefficient of determination (R^2^) great than 0.8 in each species. To calculate the ARI, the pixel intensities in each image were divided by 255, to convert them into reflectance values. Since the TopView system does not use the same wavelengths as the ARI, we used the green and red image, taken under the peak at 516 and 664 nm, the closest spectral images to the wavebands suggested for ARI (**Figure 1**).

Steele et al. (2009) introduced the modified anthocyanin content index (mACI):


(4) $$\text{mACI}={\text{R}}_{940}/{\text{R}}_{530}$$

where R is reflectance, and the subscript indicates the wavelength. Due to the availability of spectral images from the TopView system, we calculated mACI using the NIR and green image taken under the peaks at 861 and 516 nm (**Figure 1B**).

Gitelson et al. (2006) presented the modified anthocyanin reflectance index (mARI):


(5) $$\text{mARI}=({\left({\text{R}}_{530-570}\right)}^{-1}-{\left({\text{R}}_{690-710}\right)}^{-1})\times {\text{R}}_{\text{longer}\;\text{than}\;760}$$

where R is reflectance and the subscript indicates the range of wavelengths. The ranges in Eq. 5 represent the green, red, and near infrared spectrum, respectively. Due to the limitations of the TopView system, images taken under the LEDs with peaks at 516, 664, and 861 nm were used to calculate mARI.

Gamon and Surfus (1999) proposed the Red : Green ratio index (RGI) for anthocyanin prediction:


(6) $$\text{RGI}=({\text{R}}_{600-699})/({\text{R}}_{500-599})$$

where R is reflectance and the subscript indicates the range of wavelengths. The RGI was calculated from the green and red images taken under the LEDs with peaks at 516 and 664 nm (**Figure 1**). The RGI was correlated with anthocyanin concentration (R^2^ = 0.92) in *Quercus agrifolia*.

A hand-held anthocyanin content meter (ACM-200 plus; Opti-Science, Inc., Hudson, NH, USA) was also used to measure the anthocyanin content index (ACI). This meter measures absorptance in the green (peak at 530 nm) and NIR (peak at 931 nm) to estimate anthocyanin concentrations (van den Berg and Perkins, 2005), likely with similar results as the mACI.

### 2.4 Automated image analysis

We wrote a program in Python (v. 3.8) using the OpenCV library (v. 4.5.4) to process multispectral images and calculate indices for anthocyanin content (see **Script S1**). The program reads monochrome images taken under different LED spectrums and extracts the intensity of each pixel from those images (0 to 255). The pixel intensities were then used to calculate anthocyanin content indices directly or after converting the pixel intensities into reflectance values by dividing by 255. The program creates a two-dimensional matrix, containing x- and y- coordinates and the corresponding index value. The matrix was then visualized as an index image of plant objects after background removal using intensity-based thresholding, followed by despeckling. The program generates a histogram, an average, and a standard deviation of all index values from only plant objects. The standard deviation is a measure of anthocyanin variability within the plant objects.

### 2.5 A low-cost phenotyping system for anthocyanin prediction

**Figure 2:**
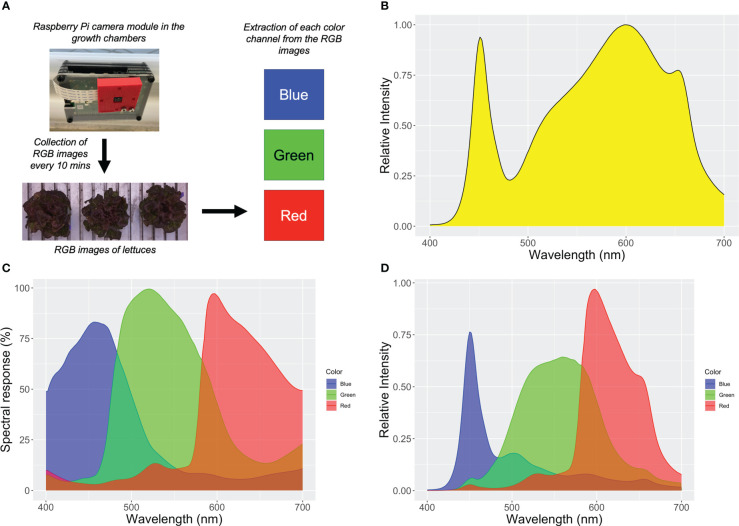
Schematic representation of acquisition process of green and red spectral images by the Raspberry Pi Camera Module V2.1 **(A)**, the normalized spectrum of the warm-white LED light fixtures in the growth chambers **(B)**, the spectral sensitivity of the camera module (Pagnutti et al., 2017) **(C)**, and the combination of the spectral sensitivity of the camera and the light spectrum in the growth chambers, showing the spectral sensitivity during acquisition of each spectral image **(D)**.

To evaluate the feasibility of using the NDAI for predicting anthocyanin concentration in a cost-effective manner, a Linux-based microcomputer (Raspberry Pi 4 model B, Raspberry Pi Foundation, Cambridge, UK) and an RGB camera (Raspberry Pi Camera Module v2.1, Raspberry Pi Foundation, Cambridge, UK) were used. The low-cost and automated imaging systems were installed in the growth chambers during the temperature/exposure time study to acquire top-view images of the lettuce plants at each harvest. Color images were taken every 10 minutes through the ‘Crontab’ command in Terminal in Raspberry Pi OS, which enables scheduling of a periodic task such as running a Python script at a given interval. The Python script for the camera operation was from the website of the Raspberry Pi Foundation (projects.raspberrypi.org/en/projects/getting-started-with-picamera) (**Figure 2A**). No gray-scale, or other calibration was used for this camera, to keep the method as simple as possible. The light spectrum in the growth chambers, spectral responses of the RGB camera, and combination of the spectral sensitivity of the RGB camera and the light spectrum in the growth chambers are visualized in **Figure 2**. The combination of them estimates the spectral response of each color channel during image acquisition under this particular light spectrum. Peak sensitivities of the system in the blue, red, and green color channels were 450, 560, and 597 nm, respectively. The spectral response of each color channel was broad compared to the LED spectrum in the TopView system. The red, green, and blue channels all have at least some sensitivity across the entire 400 – 700 nm range (**Figure 2**).

The images were analyzed with a modified program of the multispectral image analysis program that reads RGB images and extracts the pixel intensity of each color channel (see **Script S2**). The plant segmentation was established based on the red to blue ratio that showed strong contrast in the pixel intensity between plant objects and the background. The pixel intensity of plants in the blue was lower than that in the red, due to the higher absorptance in the blue spectrum. The background (metallic bottom of the growth chamber) did not show such differences in pixel intensity in the blue and red channels. With intensity-based thresholding based on the red to blue ratio, the program could separate plant objects from background and binary images were created. These binary images were then processed using erosion and dilation to improve plant segmentation. The algorithm for this segmentation will likely need to be modified for images taken under different conditions. The pixel intensity values of the pixels representing plants and for each color channel were then used to calculate NDAI. The NDAI from the RGB images was compared to the anthocyanin concentrations of the top layer of the lettuce plants. The canopy NDAI from the RGB images also was compared with the canopy NDAI from multispectral images to assess the performance of the low-cost anthocyanin phenotyping system.

### 2.6 Statistical analyses

The performance of NDAI for anthocyanin prediction was evaluated by comparing it with other reflectance indices derived from multispectral images of leaf discs and plant canopy and the corresponding anthocyanin concentrations. Anthocyanin indices of leak discs, derived from equations 1 and 3 to 6, and ACI from the hand-held meter were compared with the measured anthocyanin concentrations. The coefficient of determination (R^2^), root mean square error (RMSE), and Akaike information criterion (AIC) were calculated from the regression analyses between the indices and anthocyanin concentrations (R Core Team, 2022).

In general, an R^2^ value higher than 0.7 is considered indicative of a good model that can explain significant amount of variance, while a higher value indicates a better model fit (Frost, 2019). A lower RMSE or AIC value indicates a better model fit, but the absolute value of RMSE or AIC is not informative in determining whether a particular value indicates a good model fit (Baguley, 2018). Following the criteria of a better model in these statistical metrics, we selected the best performing model for prediction of anthocyanin concentration in the leaf discs.

Using the best-performing index model, we also tested its feasibility in prediction of anthocyanin concentration of a canopy layer using the multispectral images acquired by the TopView system and RGB images taken by the Raspberry Pi-RGB camera imaging system. To evaluate the correlation between the canopy anthocyanin concentration and the index values derived from each imaging system, we calculated R^2^ and RMSE.

## 3 Results

### 3.1 Evaluation of anthocyanin content indices

**Figure 3:**
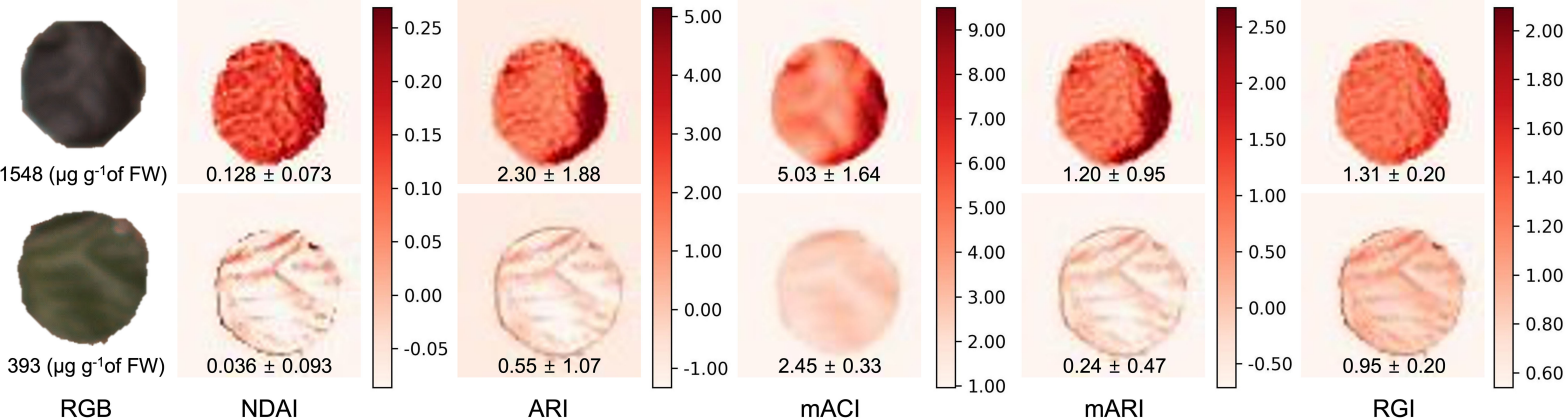
Images of various indices of anthocyanin concentration based on multispectral images. The leaf disc at the top had a higher anthocyanin concentration than the bottom one. From left to right: RGB (color) image, normalized difference anthocyanin index (NDAI; Eq. 1), anthocyanin reflectance index (ARI; Eq. 3), modified anthocyanin content index (mACI; Eq. 4), modified anthocyanin reflectance index (mARI; Eq. 5), and red to green ratio index (RGI; Eq. 6). The values in each image represents the average ± standard deviation of the corresponding index value, while the RGB images show the anthocyanin concentration of the leaf discs.

Anthocyanin concentrations of the 50 leaf discs ranged from 108 to 1673 µg g^-1^ of fresh weight. Higher anthocyanin concentrations of the leaf discs coincided with higher values of all image-derived indices, and can be observed within the false color images representing each index (**Figure 3**). The anthocyanin concentrations in these example images differed by a factor of 3.94 and the range of values obtained from the various indices differed greatly. Although the average RGI of the high-anthocyanin leaf discs was higher than that of the low-anthocyanin leaf discs, the difference was relatively small (1.38×). For the other indices, the index values for the low- and high-anthocyanin leaf discs differed by 2.05 to 5.00× (**Figure 3**).

These index images show that the distribution of anthocyanins within leaf discs was not uniform (**Figure 3**), although the leaf discs were small (3.1 cm^2^). Especially, the NDAI, ARI, and mARI images were able to display non-uniformity that was not visible in the corresponding RGB image. On the other hand, the mACI and RGI image had a somewhat limited depiction of the heterogeneous anthocyanin distribution in the leaf discs, resulting in a low coefficient of variation. The coefficient of variation in the mACI and RGI images was 0.33 and 0.15 for the higher anthocyanin concentration leaf disc and 0.13 and 0.21 for the lower anthocyanin concentration leaf disc, respectively. Images of the other indices, had coefficients of variation of 0.57 to 0.82 and 1.94 to 2.58, in the higher and low anthocyanin concentration, respectively.

**Figure 4:**
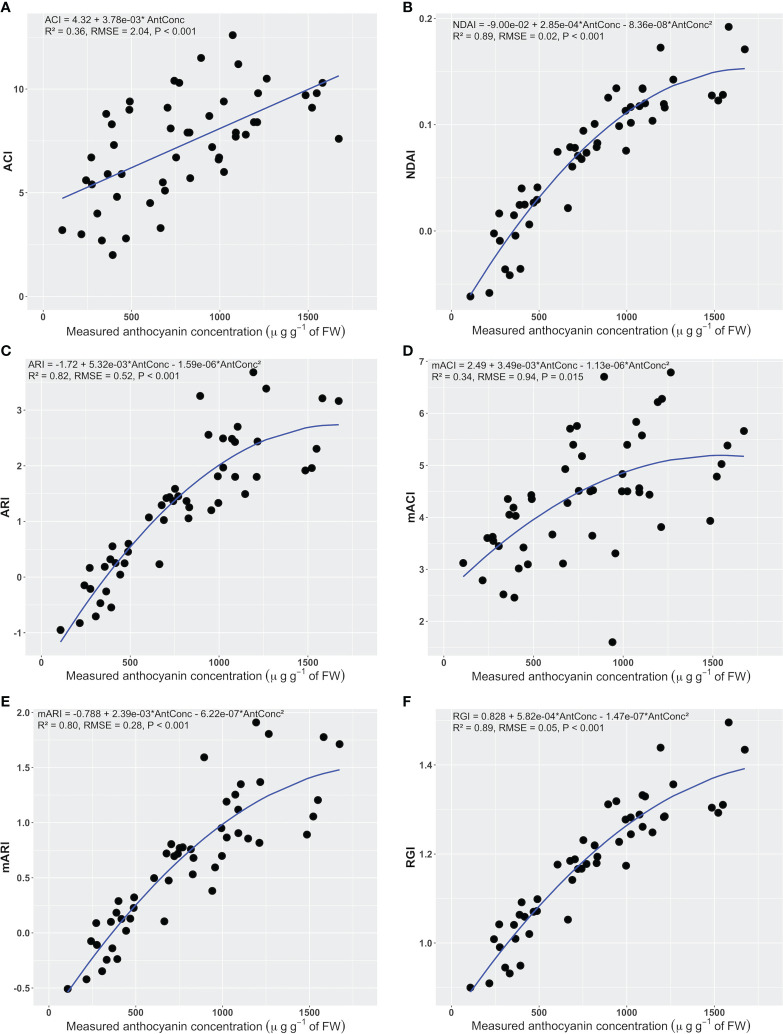
The relationship between the anthocyanin concentration of leaf discs and the corresponding anthocyanin content index of a hand-held anthocyanin content meter (ACI) **(A)**, anthocyanin content index **(B)**, anthocyanin reflectance index (ARI) **(C)**, modified anthocyanin reflectance index (mARI) **(D)**, modified anthocyanin content index (mACI) **(E)**, and red to green ratio index (RGI) **(F)** (n = 50). The regression summaries include the regression equation, coefficient of determination, root mean square error, and p-value. The blue curves show the regression equations.

An increase in anthocyanin concentration was associated with a linear or quadratic increase in all anthocyanin indices (**Figure 4**). As anthocyanin concentrations of the leaf discs increased from 108 to 1673 µg g^-1^ of FW, the average NDAI, ARI, mACI, mARI, and RGI ranged from -0.062 to 0.192, -0.950 to 3.680, 1.600 to 6.786, -0.508 to 1.909, and 0.900 to 1.496, respectively.

The NDAI, ARI, mARI, and RGI (**Figure 4** and **Table 1**) had higher R^2^ than other indices (0.80< R^2^< 0.89). These indices also had lower RMSE and AIC values (0.02< RMSE< 0.52, -237.6< AIC< 82.1). The NDAI had the highest R^2^ (0.89), lowest RMSE (0.02) and lowest AIC (-237.6) among the indices for predicting anthocyanin concentration. At the same time, the RGI had R^2^ of 0.89, RMSE of 0.05, and AIC of -149.7. The ACI and mACI resulted in lower values of these evaluation metrics (**Figure 4** and **Table 1**), resulting in R^2^ values of 0.36 and 0.34, RMSEs of 2.04 and 0.94, and AICs of 217.4 and 140.8 (**Table 1**), respectively. Changing the units of anthocyanin concentration to an area-based unit (mg m^-2^) did not affect the trends in the statistical metrics.

**Table 1:** Statistical summaries of various indices for anthocyanin content (n = 50).

Index	R^2^	RMSE	AIC	*p*-Value
ACI	0.36	2.04	217.4	< 0.001
NDAI	0.89	0.02	-237.6	< 0.001
ARI	0.82	0.52	82.1	< 0.001
mACI	0.34	0.94	140.8	0.015
mARI	0.80	0.28	19.6	< 0.001
RGI	0.89	0.05	-149.7	< 0.001

ACI, Anthocyanin content index measured by hand-held anthocyanin content meter; NDAI, normalized difference anthocyanin index; ARI, anthocyanin reflectance index; mACI, modified anthocyanin content index; mARI, modified anthocyanin reflectance index; and RGI, red and green ratio index.

Differences in the statistical metrics can be used to determine which wavelengths to use for an anthocyanin index. The NDAI, ARI, and RGI all use reflectance in the green and red, while mARI also uses NIR, whereas ACI and mACI use reflectance in the green and NIR. This suggests that the use of the red waveband is preferable to the use of NIR, likely because the red reflectance can help account for different chlorophyll concentrations.

The NDAI predicted anthocyanin concentration slightly better than the RGI, based on its lower AIC value and RMSE (**Table 1**). Based on these results, the best index for image-based anthocyanin phenotyping is NDAI, while RGI also performs well. Note that all indices, except for ACI, had a non-linear relationship with the measured anthocyanin concentration. Interestingly, the commercially-available anthocyanin meter, which measures ACI, performed poorly (R^2^ = 0.36).

### 3.2 Canopy NDAI imaging

**Figure 5:**
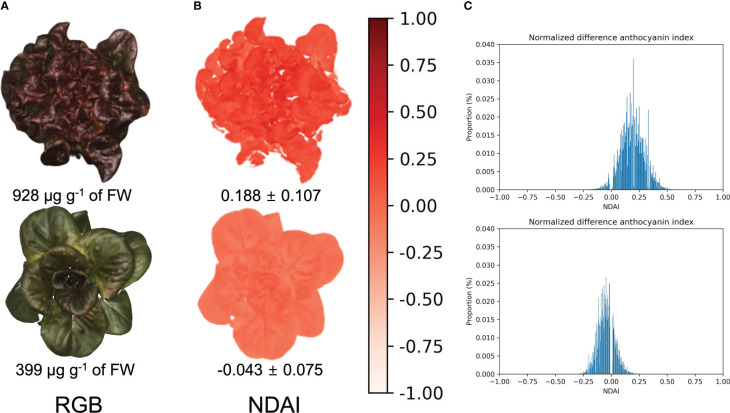
Canopy RGB **(A)** and normalized difference anthocyanin index (**B**; NDAI) images of red lettuce cultivars ‘Rouxai’ (top) and ‘Teodore’ (bottom) and their corresponding histograms **(C)**. The scale bar on the right side represents the NDAI values within these NDAI images. Values below the NDAI images represent mean ± standard deviation of NDAI within the NDAI images, while values below the RGB images represent the anthocyanin concentration.

Canopy NDAI images were obtained by using the pixel intensities from canopy images taken under red and green light (**Figure 5**). Canopy NDAI images of ‘Rouxai’ and ‘Teodore’ clearly showed the difference in their anthocyanin concentrations (**Figure 5C**). The canopy ACI images provide spatial information by depicting the distribution of anthocyanins within the canopy.

**Figure 6:**
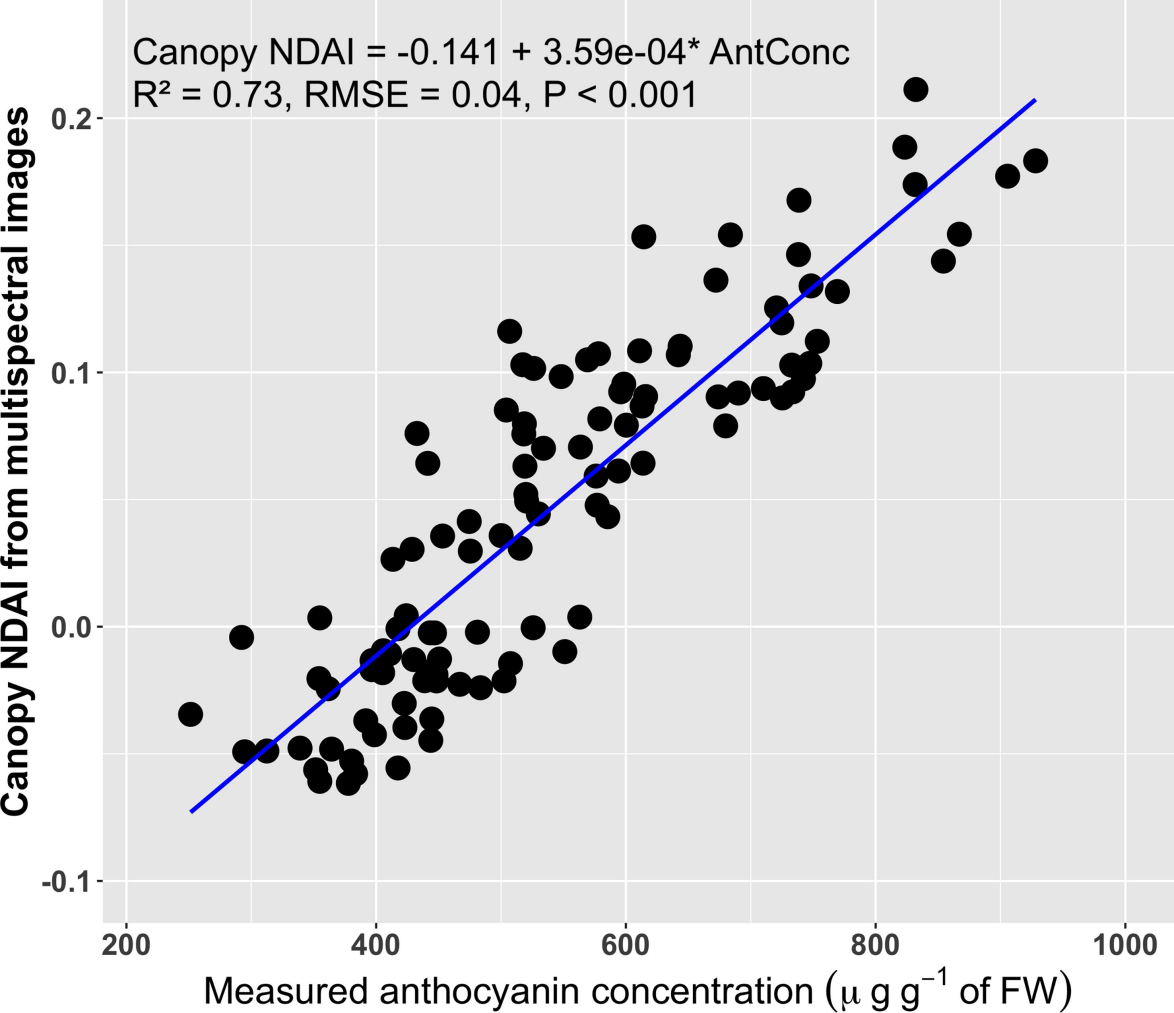
Correlation between the canopy normalized difference anthocyanin index (NDAI, averaged over the entire part of the canopy visible in the images) derived from multispectral images and the anthocyanin concentrations of the top layer of the canopy (n = 108). The blue line is the regression line.

The anthocyanin concentrations of top canopy layer the red lettuce cultivars ranged from 251 to 928 µg g^-1^ of FW, while the average canopy NDAI increased from -0.062 to 0.211 with increases in the anthocyanin concentrations (**Figure 6**). Anthocyanin concentrations in the top canopy layer had a positive correlation (R^2^ of 0.73 and RMSE = 0.04) with the average canopy NDAI. This relationship was not a strong as that for the leaf discs, likely because of the non-uniform distribution of the anthocyanins. Anthocyanins were extracted from the top layer of the canopy, but it was not possible to sample the exact same part of the canopy that was visible in the images. However, the high R^2^ and low RMSE of the canopy NDAI versus extracted anthocyanin concentration indicate that this method is useful to predict anthocyanin concentrations using canopy multispectral images.

### 3.3 A low-cost imaging system for canopy NDAI

The red and green pixels of the RGB camera under the white LED fixtures in the growth chambers were used as proxies for the green and red images from the TopView multispectral imaging system. The green and red channels of the TopView system had peaks at wavelengths of 516 and 664 nm, respectively (**Figure 1**). The red channel of the RGB camera had a sensitivity of >50% at wavelengths from 580 to 700 nm with a peak at 596 nm, but was also sensitive to wavelengths below 580 nm (**Figure 2B**). The green channel had a sensitivity above 50% at wavelengths from 477 to 595 nm with a peak at 521 nm (**Figure 2B**). Because of the different spectral responses of the RGB camera and multi-spectral imaging system, we tested whether the canopy NDAI from the multispectral images and RGB camera are correlated. Given the different spectral response of the two systems, we did not expect the values to be identical.

**Figure 7:**
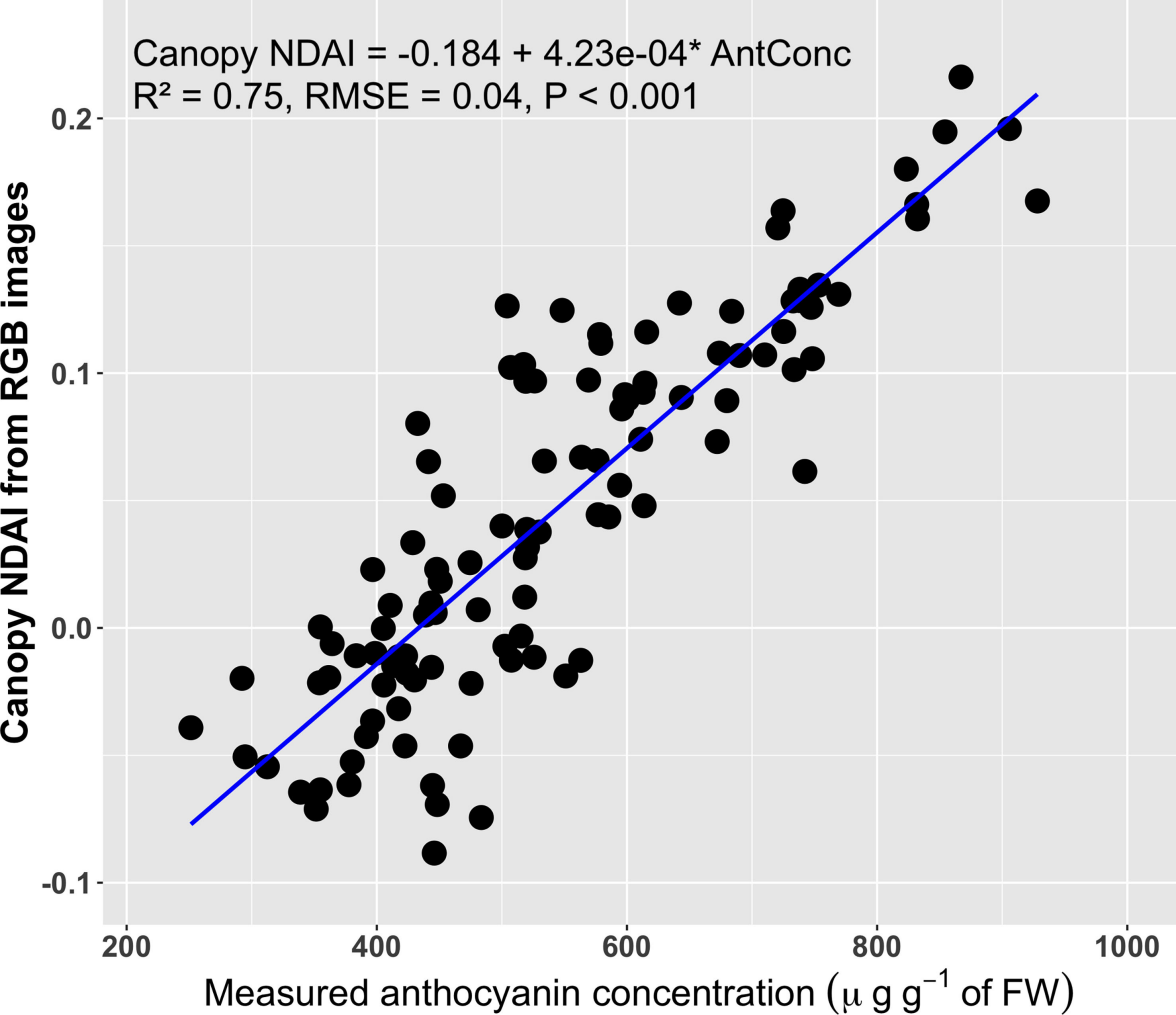
Correlation between the normalized difference anthocyanin index (NDAI) derived from RGB images and averaged over the entire visible part of the canopy and the canopy anthocyanin concentration (n = 108) **(A)**. The blue line is the regression line.

The average canopy NDAI based on the RGB images was positively correlated with the anthocyanin concentration (R^2^ = 0.75, RMSE = 0.04; **Figure 7**). The average canopy NDAI from the RGB images ranged from -0.088 to 0.216, similar to the range of the canopy NDAI from the multispectral images, which ranged from -0.061 to 0.211.

**Figure 8:**
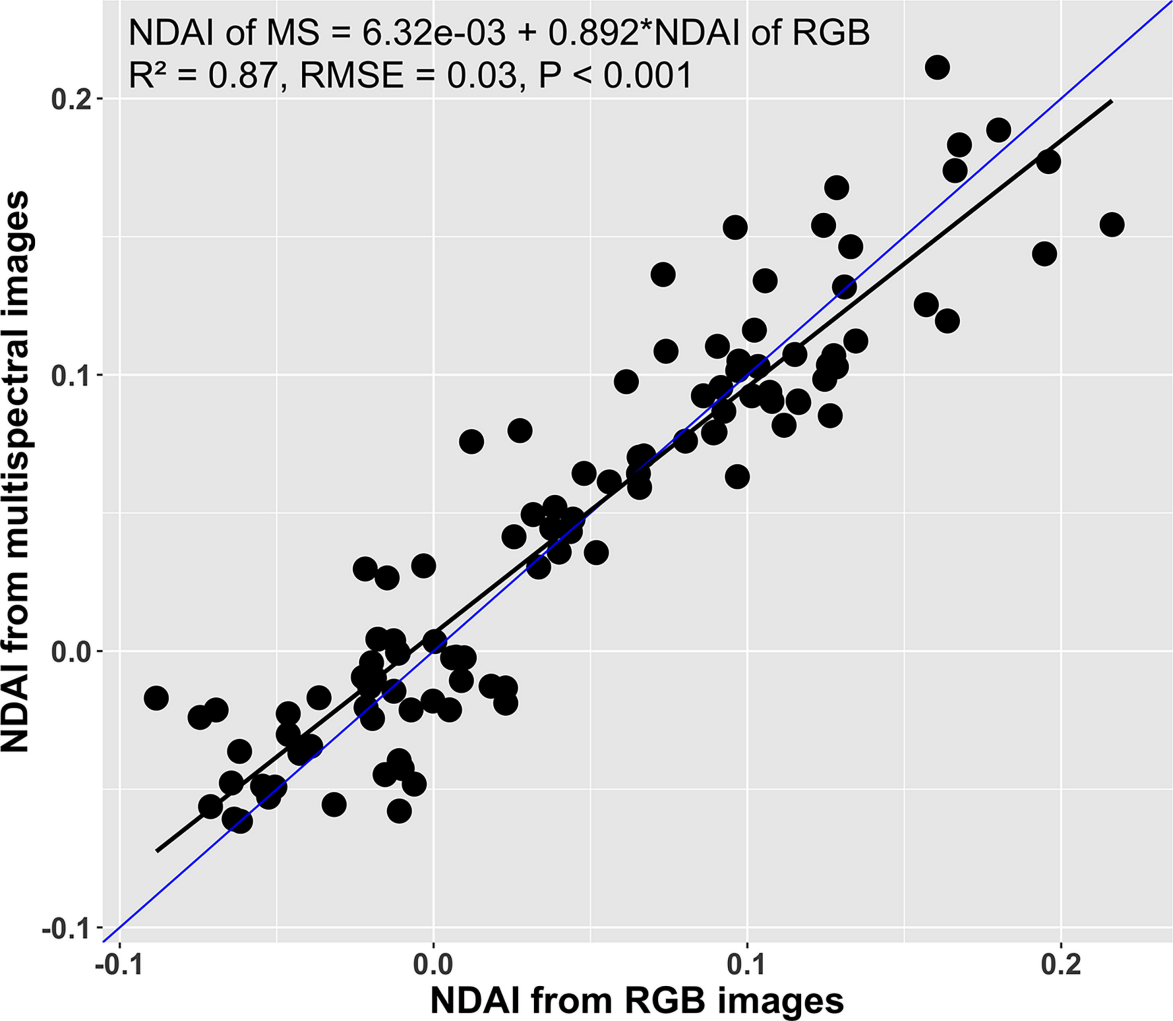
Correlation between canopy normalized difference anthocyanin index (NDAI) derived from multispectral and RGB images (n = 108). The black line is the regression line, while the blue line is a 1:1 relation line.

Likewise, the canopy NDAI from the multispectral images and from the RGB images were positively correlated (R^2^ = 0.87, RMSE = 0.03; **Figure 8**). Comparing the regression model (black line) to the 1:1 line (blue line) suggests that the NDAI from RGB images was slightly overestimated at NDAI values< 0.05 and underestimated at NDAI > 0.05. Such differences might be associated with errors in background removal during the RGB image processing, differences in the light spectrum during the RGB image acquisition, angle of the camera, and spectral sensitivity of the two methods. Despite these differences between the two imaging approaches, there was a strong correlation between the two methods, indicating that the RGB images can be used to estimate NDAI.

## 4 Discussion

Evaluation of the image-derived anthocyanin indices demonstrates the feasibility of image-based phenotyping for estimating anthocyanin concentrations in lettuce. Our initial testing was conducted using leaf discs with the multispectral imaging system. Because using a narrow area of leaf and a reliable spectral imaging system can be considered a reference condition for development of a precise prediction model for anthocyanin concentration. The selected NDAI, therefore, resulted in R^2^ of 0.73 for the correlation between the canopy anthocyanin concentration and the canopy NDAI using the multispectral imaging system. Furthermore, the canopy NDAI derived from the RGB images resulted in R^2^ of 0.75, demonstrating that a potential method of whole plant or crop anthocyanin phenotyping at a low cost.

In addition, NDAI values obtained from the multispectral imaging system and RGB images were similar, indicating that a regular digital camera, combined with a broad-band white light source may be all that is required for NDAI imaging. All tested anthocyanin indices showed a positive relationship with leaf disc anthocyanin concentrations, but the performance of the different indices varied greatly (**Figure 4** and **Table 1**). Although our multispectral images (**Figure 1**) do not use the same wavebands as those typically used for ARI, mARI, and mACI (Eq. 3-5) (e.g. ARI and mARI required 700 nm while we used 664 nm and mACI required 940 nm while we used 861 nm) (Gitelson et al., 2006; Gitelson et al., 2009; Steele et al., 2009), the image-derived indices that used the green and red part of the spectrum (NDAI, ARI, mARI, and RGI) achieved a reasonably good model fit in prediction of anthocyanin concentrations (R^2^ ≥ 0.8, RMSE ≤ 0.52, AIC ≤ 82.1).

That success is associated with the role of the green and red spectrum in the anthocyanin indices. The variation in reflectance in the green part of the spectrum (510 to 550 nm) is partly determined by anthocyanin concentration, because anthocyanins absorb green photons effectively (Neill and Gould, 2000; Gitelson et al., 2001). However, chlorophyll also absorbs green light. Reflectance in the red part of the spectrum (660 nm to 710) can account for variability in chlorophyll concentration due to the relationship between chlorophyll concentration and absorptance in this part of the spectrum (Lichtenthaler, 1987; Gitelson et al., 1996). The NDAI, ARI, mARI, and RGI were able to quantify anthocyanin concentration accurately by removing interference from variable chlorophyll concentrations (Gitelson et al., 2009).

Not all indices attempt to correct for the influence of chlorophyll. The ACI and mACI, use the green and NIR part of the spectrum, resulting in R^2^ values of 0.36 and 0.34, respectively. This was consistent with the report by Steele et al. (2009), who reported a weak relationship between mACI and anthocyanin content of grape vine leaves (R^2^ = 0.06). Slaton et al. (2001) reported that the reflectance in the NIR is associated with characteristics of leaf structure, such as leaf thickness, rather than chlorophyll content. Therefore, using NIR instead of red has no benefits in accounting for chlorophyll. The mARI uses NIR as a third waveband to account for variability in leaf thickness and light scattering within the leaf (Gitelson et al., 2006), but this did not have any distinctive advantages over the indices using only green and red, such as NDAI, ARI and RGI based on statistical metrics.

The form of the index equation determines the range of values the index can have. NDAI, adopting the NDVI equation, is constrained to the range between -1 and 1, but in reality ranged only from -0.25 to 0.5 (**Figure 5**). The other indices do not have constrained ranges for their value. This could potentially result in extreme values, making averaging of multiple readings potentially meaningless. Since the NDAI had slightly better statistical performance than RGI and the other indices (**Table 1**) and is constrained between -1 and 1, we conclude that NDAI has advantages over the other indices.

There are many successful examples of anthocyanin predictions based on hyperspectral imaging combined with machine learning techniques (Chen et al., 2015; Askey et al., 2019; Simko, 2020; Cho et al., 2021; Kim et al., 2021). However, these machine learning models require hyperspectral imaging systems with similar wavelengths as used to develop the machine learning model. Thus, such models may not perform well when applied to data collected with different hyperspectral imaging systems or under different lighting conditions. In addition, hyperspectral imaging systems provide much more information than our NDAI calculation requires, and are generally expensive. The high cost of technical requirements of hyperspectral imaging and machine learning makes widespread adoption difficult.

NDAI requires only red and green images, which can be acquired from RGB images or simple multispectral imaging systems. The NARI, having the identical equation as NDAI, successfully detected variability in anthocyanin concentrations in mountain shrublands (Bayle et al., 2019). However, it uses satellite imagery as its source of spectral reflectance, and relies on narrow green and red-edge wavebands. Furthermore, the NARI has not been evaluated by comparing index values to measured anthocyanin concentrations. On the other hand, the NDAI, with the low-cost RGB imaging or the multispectral imaging, had a good correlation with canopy anthocyanin concentrations. That suggests that pictures taken by a cellular phone or any color camera, which can capture red and green color channels, can be used to calculate NDAI. Due to its simplicity and the lack of need for a narrow specific waveband, RGB-derived NDAI can be easily implemented at the single leaf or whole canopy scale, including in remote sensing and indoor horticultural production. Such a system can be built using an RGB camera and Raspberry Pi microcomputer, at a cost of about $60. The simplicity, low cost, and automated processing can make multispectral imaging available to a wide range of researchers and growers, who need such technology but cannot afford expensive systems.

Some limitations of this image-based phenotyping approach are associated with imaging of plants from above. The images largely capture the properties of the cell layers near the top of the canopy. If anthocyanins are present on the bottom side of leaves, imaging may not detect them. In addition, the camera can only see one layer of leaves and the system thus provides no information regarding lower leaves if they are obscured by the upper leaves. Segmentation of plant objects is also a prerequisite to obtain anthocyanin indices. Finally, NDAI values, as well as other indices, are affected by the spectrum of the light under which the images are taken. As long as the light spectrum is the same for all images, calculated NDAI values will still reflect differences in anthocyanin content. However, if the light spectrum changes, this poses a challenge for comparing NDAI values. This might be solved by *in situ* spectral calibration procedures using an in-scene reference card (Davies et al., 2022).

The monitoring of the anthocyanin content using NDAI has a wide range of agricultural and ecological applications. Not surprisingly, the optical properties of anthocyanins in fruit and vegetative tissues are similar. Therefore, NDAI may be used to assess ripeness of fruits that accumulate anthocyanins during ripening, such as grapes and strawberries. Indeed, Underhill et al. (2020) showed that the RGB color space differentiated variability in grape skin color, associated with different anthocyanin concentrations. Strawberries with higher concentrations of anthocyanin had lower pixel intensities in the green spectrum (the lowest intensity was near 530 nm) based on hyperspectral imaging (Cho et al., 2021). Monitoring anthocyanins in fruits and vegetables will be beneficial not only in assessing phenotypic variation in anthocyanins in a non-destructive manner, but also in automating post-harvest quality evaluation or for robotic harvesting. Finally, the integration of NDAI phenotyping and environmental control systems used in controlled environment agriculture may be used for dynamic environmental control to stimulate anthocyanin production in many vegetables and fruits.

## Data availability statement

The raw data supporting the conclusions of this article will be made available by the authors, without undue reservation.

## Author contributions

CK and MI conceived the original idea; CK and MI designed the experiment; and CK collected and analyzed the data and wrote the first draft, with inputs from MI. Both authors contributed to the article and approved the submitted version.

## Supplementary material

Document S1 Manual for image analysis script.

Script S1 Python script for normalized difference anthocyanin index using multispectral images.

Script S2 Python script for normalized difference anthocyanin index using RGB images.
