# Supplementary material for: Image-based phenotyping to estimate anthocyanin concentrations in lettuce
Source: Front Plant Sci. 2023 Apr 3;14:1155722. doi: 10.3389/fpls.2023.1155722 (PMC10109386; doi:10.3389/fpls.2023.1155722)
Supplement: Document S1 — Manual for image analysis script. [file DataSheet_1.zip › Document S1. Manual for image analysis script.pdf]

Before you start: Download & Install ANACONDA

- https://www.anaconda.com/products/individual

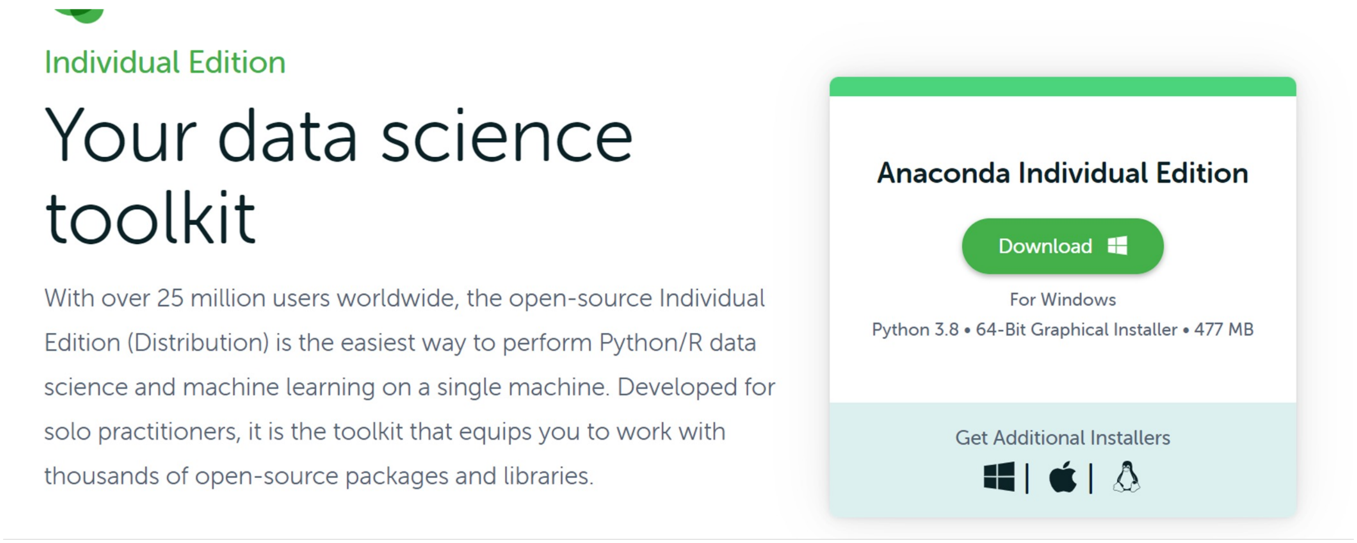

Before you start: Install OpenCV

- Search “Anaconda Prompt (Anaconda3)” after you press the ‘Window’ button

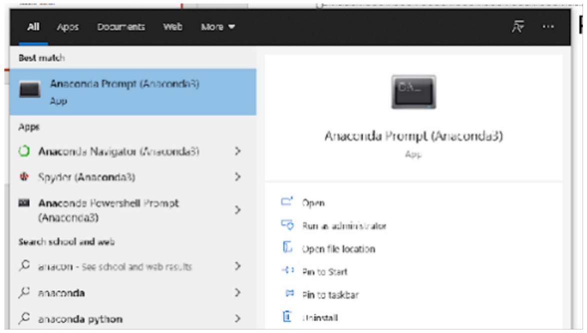

- Type “pip install opencv-contrib-python” in the prompt, which installs the OpenCV.

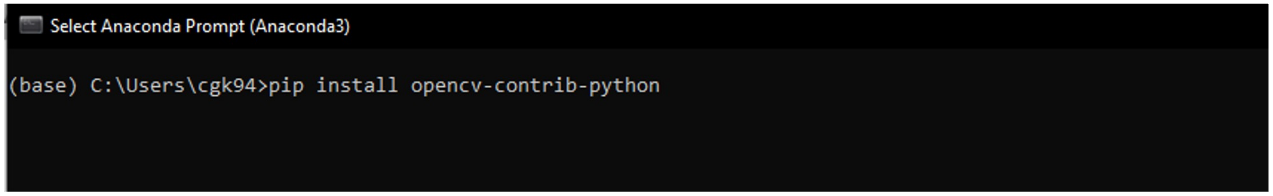

Before you start: Launch ANACONDA - Spyder

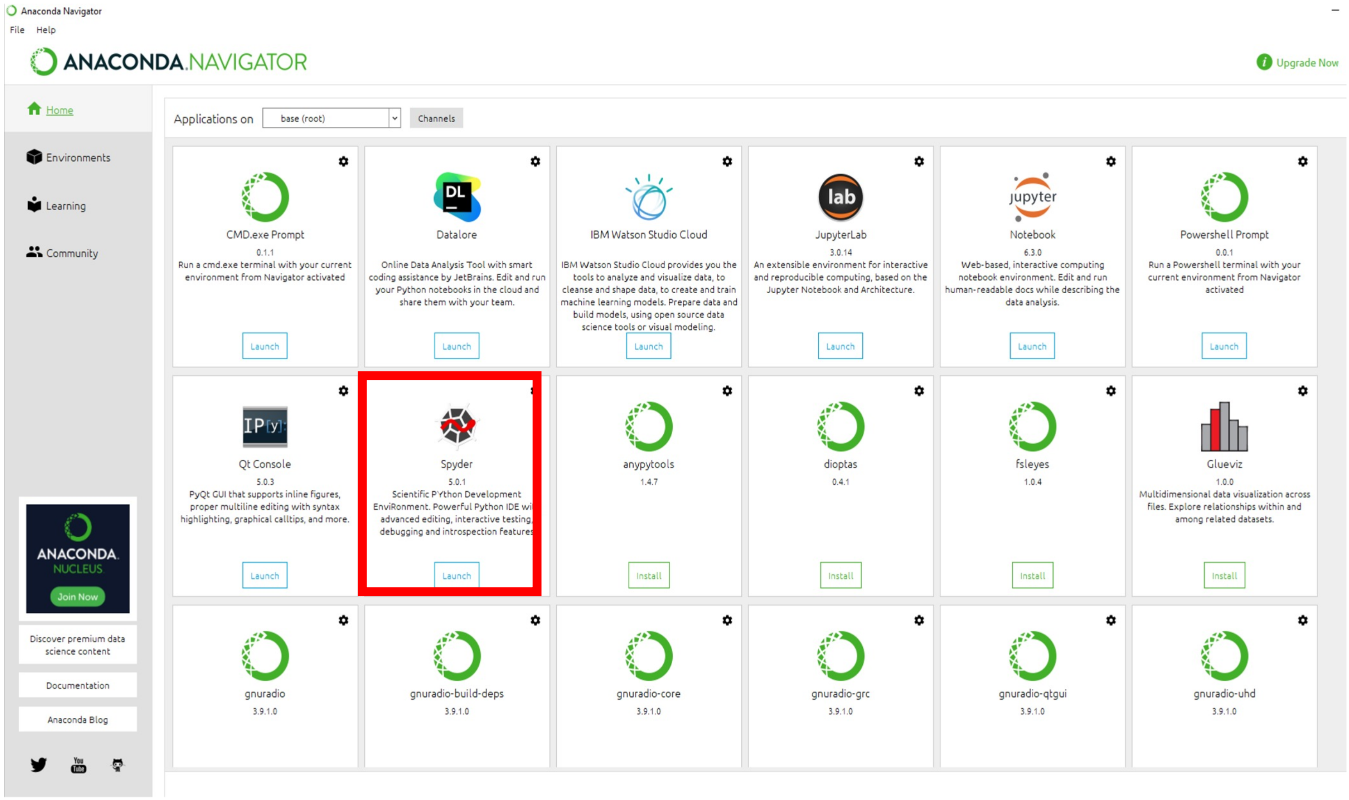

NDAI with multispectral images

- The program generates a csv file with the average and standard deviation of NDAI.
- Open ’Script S1.py’ from the Supplemental material
- Scroll down to line 98 of the script

```
96    #IEMI(minPxs, Histmin, Histmax, csvname, imageformat, folder):
97    masterpath = "/Users/cgk/Downloads/Script1 input"
98    IEMI(4000, 10, 30, masterpath+"/result.csv",'/*.png',masterpath)
```

NDAI with multispectral images

```
#IEMI(minPxs, Histmin, Histmax, csvname, imageformat, folder):
masterpath = "/Users/cgk/Downloads/Script1 input"
IEMI(4000, 10, 30, masterpath+"/result.csv",'/*.png',masterpath)
```

- The first three parameters in ‘IEMI’ function is for generating mask image using chlorophyll fluorescence image.
- User requires to define their own ‘masterpath’, which is a directory storing multispectral image sets.
- User requires to change image format to match with their multispectral images.

Example: NDAI with multispectral images

```
#IEMI(minPxs, Histmin, Histmax, csvname, imageformat, folder):
masterpath = "/Users/cgk/Downloads/Script1 input"
IEMI(4000, 10, 30, masterpath+"/result.csv",'/*.png',masterpath)
```

- Download ‘Data S1. Script1 input’ from http://bit.ly/3urP6YE
- Change ‘masterpath’ as “the_directory_of_folder/Data S1.Script1 input”
- The example outcome of the program is ‘Data S3. Script1 outcome’ in http://bit.ly/3urP6YE

NDAI with RGB images

- The program generates a csv file with the average and standard deviation of NDAI.
- Open ’Script S1.py’ from the Supplemental material
- Scroll down to line 115 of the script

```
114     masterpath = "/Users/cgk/Downloads/Script2 input"
115     NDAI_EX (1, '/*.jpg', 5000, 50, masterpath+"/result.csv",masterpath)
```

NDAI with RGB images

```
masterpath = "/Users/cgk/Downloads/Script2 input"
NDAI_EX (1, '/*.jpg', 5000, 50, masterpath+"/result.csv",masterpath)
```

- User requires to define their own ‘masterpath’, which is a directory storing multispectral image sets.
- User requires to change image format to match with their RGB images.

Example: NDAI with RGB images

```
#IEMI(minPxs, Histmin, Histmax, csvname, imageformat, folder):
masterpath = "/Users/cgk/Downloads/Script1 input"
IEMI(4000, 10, 30, masterpath+"/result.csv",'/*.png',masterpath)
```

- Download ‘Data S2. Script2 input’ from http://bit.ly/3urP6YE
- Change ‘masterpath’ as “the_directory_of_folder/Data S2.Script2 input”
- The example outcome of the program is ‘Data S4. Script2 outcome’ in http://bit.ly/3urP6YE
